# Treatment of patients with anorexia nervosa and comorbid post-traumatic stress disorder; where do we stand? A systematic scoping review

**DOI:** 10.3389/fpsyt.2024.1365715

**Published:** 2024-02-26

**Authors:** Elske van den Berg, Kirsten Pellemans, Caroline Planting, Peter Daansen, Ella van Beers, Margo de Jonge, Carolien Christ, Jack Dekker

**Affiliations:** ^1^ Novarum Center for Eating Disorders, Amstelveen, Netherlands; ^2^ Arkin Mental Health Institute, Amsterdam, Netherlands; ^3^ GGZ Ingeest VU University Medical Center Mental Health Institute, Amsterdam, Netherlands; ^4^ PsyQ Mental Health Institute, Beverwijk, Netherlands; ^5^ Department of Clinical Psychology, Leiden University, Leiden, Netherlands; ^6^ Department of Clinical Psychology, VU University, Amsterdam, Netherlands

**Keywords:** systematic review, scoping review, anorexia nervosa, post-traumatic stress disorder, comorbidity, concurrent treatment, psychological treatment, research gap identification

## Abstract

**Objective:**

Comorbid post-traumatic stress disorder in patients with anorexia nervosa may negatively affect the course of anorexia nervosa treatment, which is already challenging. There are currently no guidelines or recommendations on concurrent treatment approaches for both anorexia nervosa and post-traumatic stress disorder. This systematic scoping review aims to explore the feasibility, acceptability and effectiveness of psychological trauma-focused treatment concurrently offered to underweight patients receiving anorexia nervosa treatment.

**Method:**

A multi-step literature search, according to an *a priori* protocol was performed. Databases PubMed, Embase, APA PsycINFO, Web of Science, Scopus and Cochrane Central were searched up to September 19^th^ 2022, and the search was rerun June 19^th^ 2023. For quality assessment, *Risk of Bias in Non-randomised Studies-of Interventions* tool was used.

**Results:**

The extensive search yielded 1769 reports, out of which only three observational pilot studies, both English and German, published between 2004 and 2022, could be included. The included studies reported on a total of 13 female participants between 16 and 58 years old, with anorexia nervosa or otherwise specified feeding or eating disorder, baseline BMI ranging between 14.6 and 16.5, who received concurrent anorexia and post-traumatic stress disorder treatment. In all participants, the emotional and cognitive functioning was sufficient to process the offered trauma-focused interventions, despite their significantly low body weight.

**Discussion:**

The findings of this review identify a dearth of treatment research on knowledge of concurrent trauma-focused treatments for patients with anorexia nervosa. Refraining patients with anorexia nervosa from trauma-focused treatment may not be warranted.

## Introduction

Anorexia is a complex mental disorder with modest long-term remission rates ranging from 30% to 63% (1). Psychiatric comorbidity is reported in over 70% of individuals with anorexia nervosa (2). Among those co-occurring psychiatric illnesses, trauma-and stressor-related disorders are commonly reported (3). High prevalence rates of emotional-, physical- and/or sexual abuse and/or neglect have been found in patients with anorexia nervosa, binge-eating/purging subtype (4, 5). With rates varying between 21% to 59%, this prevalence rate is high relative to both psychiatric and to general populations (5). Lower abuse rates, however, have been reported in patients with anorexia nervosa, restrictive subtype. This difference in abuse rate between the two anorexia nervosa subtypes aligns with findings that eating disorder patients who experienced childhood maltreatment report more binge-purging behaviors (5, 6). Patients who have been exposed to childhood maltreatment report more severe eating disorder pathology, have an earlier onset of the eating disorder, and a higher rate of psychiatric comorbidity compared to eating disorder patients without a history of childhood maltreatment (5).

Childhood maltreatment is a significant risk factor for developing post-traumatic stress disorder (PTSD); however, not all patients who suffered from abuse do develop PTSD (7). Studies on the prevalence of comorbid PTSD in patients with anorexia nervosa are scarce. In a study on female adult patients with eating disorders, Tagay et al. (8) found that 23.1% of the anorexia nervosa subsample (*N* = 52) met criteria for PTSD. The PTSD diagnoses in this study were established by using a self-rating questionnaire. When an eating disorder and PTSD occur together, it is assumed that disturbed eating disorder behaviors like severe food restriction or bingeing/purging, may provide an escape and avoidance from distressing trauma-related memories, thoughts, or feelings (9, 10) and may facilitate a decrease of hyperarousal (6). Therefore, these disturbed eating disorder behaviors may perpetuate both PTSD and the anorexia nervosa, leading to a cyclical state of relying on eating disorder behaviors to soothe and manage PTSD symptoms. It is suggested that maladaptive emotion regulation strategies mediate the relationship between eating disorder pathology and PTSD (11, 12).

Findings are inconclusive on whether comorbid PTSD negatively affect treatment outcomes for patients with anorexia nervosa (3, 13). This inconclusiveness is likely in part due to the wide variety of included participants and diagnostic criteria used in the study samples (3). It has been established however that, across eating disorder samples, co-occurring PTSD is associated with higher dropout rates from treatment (3, 13).

Despite the relatively high prevalence of PTSD seen in eating disorder patients, no guidelines or recommendations are available on how to support patients with this comorbidity (14–16). Patients with severe mental illnesses are frequently refrained from trauma-focused treatment as symptom exacerbation and relapse are feared (17). In addition, eating disorder patients with a low BMI are commonly refused specialized trauma treatment (18) as malnutrition is assumed to have impaired their emotional and cognitive functioning which might hinder patients’ ability to engage in trauma treatment. Consequently, patients with anorexia nervosa who are unable to regain weight out of fear that distressing trauma-related memories, thoughts, or feelings may intensify when starvation stops, are often deemed ineligible for specialized trauma treatment. Importantly, however, findings on this assumed impairment are inconclusive. Therefore, trauma treatment should not be delayed until a healthy weight has been reached (18, 19). Refraining patients from trauma-focused treatments puts patients at risk of developing a chronic status of both anorexia nervosa and PTSD and of multimorbidity (18), leading up to high treatment costs. Therefore, developing concurrent treatment approaches for those underweight eating disorder patients with PTSD who are at risk of getting locked-in, is critical and has been identified as a current priority by eating disorder health professionals, those with lived experiences and by affected families (20). Emphasizing the importance of expanding treatment research on anorexia nervosa is not new: more than two decades ago, leading experts in the field already highlighted the urgency of research addressing comorbidity in patients with anorexia nervosa (21).

Recently, some studies on concurrent psychological treatments for eating disorders and PTSD have been published. However, patients with anorexia nervosa were not included as participants were not eligible if they had a significantly low body weight; in a randomized controlled trial by Trottier and colleagues, a BMI ≥ 18.5 was required in order to be included in the study (22). In a study by Claudat and colleagues, patients were eligible with a minimum weight of 85% Ideal Body Weight (23).

This present systematic scoping review provides an up-to-date overview of the feasibility, acceptability and effectiveness of concurrent trauma-focused treatments offered to patients with anorexia nervosa and a significantly low body weight, who are receiving eating disorder treatment. Findings from this review will provide a greater understanding of concurrent treatment approaches offered to moderate, severe and extreme underweight patients. This review will contribute to designing optimal concurrent treatment interventions of which the efficacy can be examined in further trials.

## Method

This systematic review was conducted in accordance with the Preferred Reporting Items for Systematic Review and Meta-Analysis Protocols (PRISMA-P) guidelines (24). The protocol for this review was registered with the International Prospective Register of Systematic Reviews under registration number CRD42022353707.

### Search strategy and selection procedure

A systematic extensive electronic database literature search from 1946 up to September 19^th^ 2022 was conducted in databases PubMed, Embase, APA PsycINFO, Web of Science, Scopus, and Cochrane Central and was rerun June 19^th^ 2023. To search for relevant reports, the following concepts were combined: “Anorexia nervosa” AND “therapy” AND “Stress disorders, Traumatic”. See Appendix A for the full elaborated search string. Articles were also searched via hand searches of reference lists by the first two authors. The World Health Organization’s International Clinical Trials Registry was searched for unpublished studies. The literature search and study selection were conducted independently by the first two authors. After screening titles and abstract, these two authors independently applied eligibility criteria in the full text evaluation. When needed, both authors discussed inclusion until consensus was reached. The software used for recording decisions was Rayyan.

### Selection criteria

Studies could be included when written in English or German, and published in peer-reviewed journals, book chapters or congress papers. As this review concerns an under-researched area, no restrictions were set on type of research design.

### Participants

Studies were eligible when both criteria were met:

(a) patients were diagnosed with anorexia nervosa; with eating disorder not otherwise specified with underweight status according to DSM-IV criteria; or with other specified feeding or eating disorder with significantly low body weight according to DSM-5 criteria.

With regard to the level of severity of baseline body mass index, no minimum was set.

(b) patients were diagnosed with PTSD.

### Interventions

Studies were included when both the anorexia nervosa treatment and PTSD treatment involved at least some in-person, verbal contact. Both treatments had to consist of psychological interventions targeted at diminishing anorexia nervosa pathology and PTSD pathology. Both treatments had to be provided simultaneously. With regard to the way of deliverance of the interventions, no restrictions were set. Non-psychological interventions such as repetitive transcranial magnetic stimulation treatment or infra-low frequency neurofeedback were not included.

### Comparator & setting

With regard to both possible control conditions and treatment settings, no restrictions were set.

### Outcomes

Studies were eligible if, before and after the offered interventions, treatment outcomes on anorexia nervosa pathology and on PTSD pathology were described.

### Time frame

Due to the expected limited number of eligible studies, only pre- and post-treatment measurements were included, and no data on follow-up were required. If only follow up data were reported and no end-of-treatment data were available, follow up data were included and used as post-treatment measure.

### Data extraction

Data extraction was performed independently by the first two authors. Any disagreements were discussed until consensus was reached. From the included studies, the following data were extracted: study design; number of participants; gender, age and baseline BMI of participants; anorexia nervosa subtype; presence of additional psychiatric diagnoses; type, dose and duration of anorexia nervosa treatment offered; type, dose and duration of trauma-focused interventions offered; measurements used to assess treatment effects; end-of-treatment effects on both anorexia nervosa and PTSD pathology; findings on the ability of participants to process the trauma-focused interventions; occurrence of (serious) adverse events; patient responses to the therapies offered.

Missing data were handled by reaching out to first authors of the studies from which data are missing.

### Quality assessment

As it was expected that mainly non-randomized studies would be included in this review, the ‘Risk of Bias in Non-randomised Studies-of Interventions’ tool (ROBINS-I; 25) was used to assess their quality. In this tool, the domains through which bias might be introduced are (a) confounding, (b) participants selection, (c) classification of interventions, (d) deviations from intended interventions, (e) missing data (f) measurement of outcomes and (g) selection of reported results. Per study, an overall risk of bias judgement was reached with “low”, “moderate”, “serious” or a “critical” risk of bias (see ROBINS-I; 25). The first and second author independently applied the tool, and the final rating was reached through consensus.

## Results

**Figure 1:**
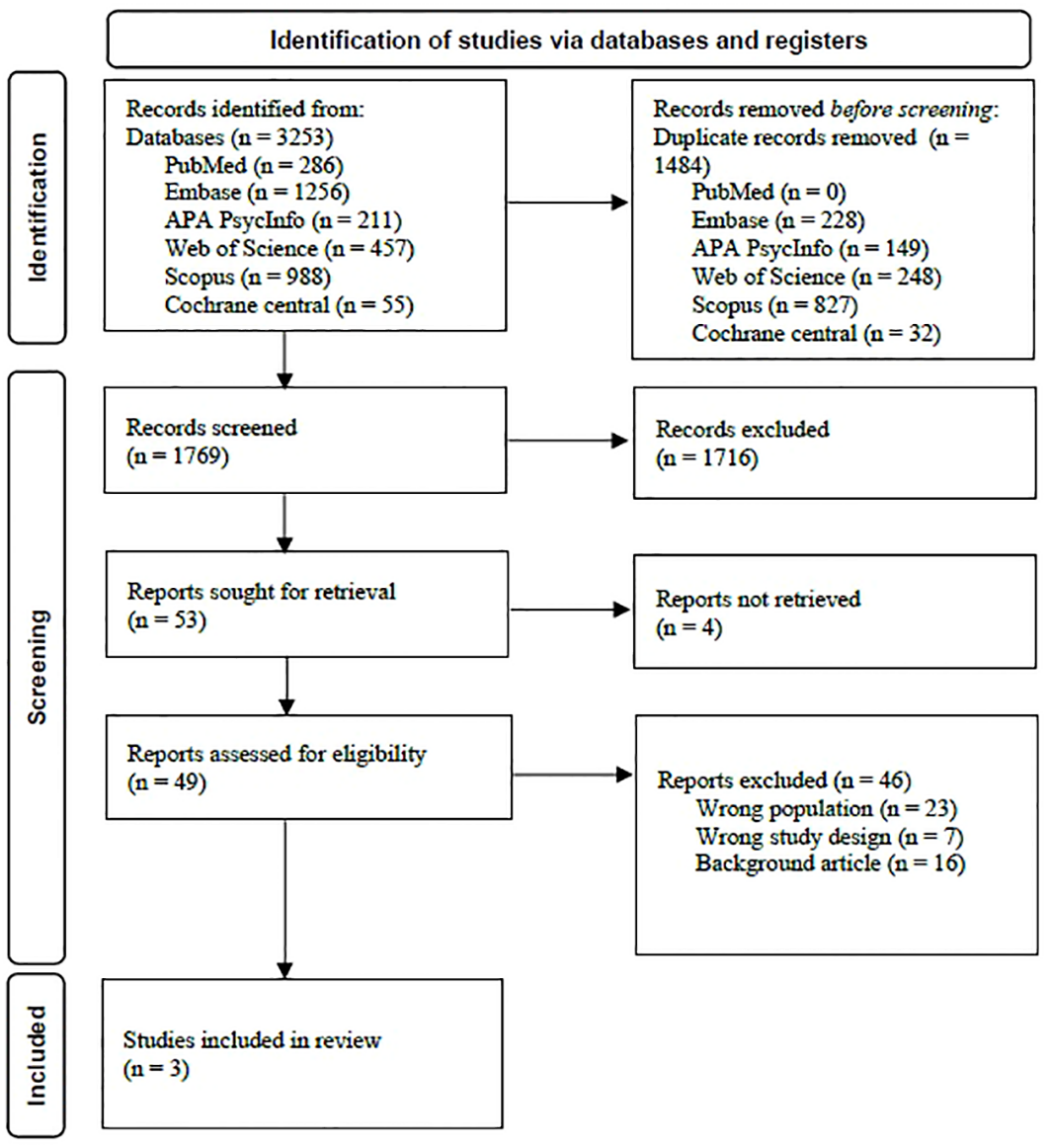
Study identification and selection (PRISMA flow chart).

The search for concurrent treatment approaches for anorexia nervosa and PTSD yielded 1769 results, out of which three studies on 13 eligible participants were included. Please see **Figure 1** for study identification and selection of the searches.

### Study characteristics

The included studies were two case series (*N* = 10; 26) and (*n* = 2; 27) and one case report (28), published between 2004 and 2022. Two studies took place in Europa (Svaldi study in Germany and Ten Napel-Schutz study in the Netherlands) and one study took place in the USA. A concurrent PTSD treatment was offered to a total of 13 female participants, aged between 16 and 58 years with a baseline BMI ranging from 14.6 to 16.5. One participant was diagnosed with other specified feeding or eating disorder, three participants were diagnosed with anorexia nervosa, binge-eating/purging subtype and nine participants were diagnosed with anorexia nervosa, without a specified subtype. Two studies took place within an inpatient eating disorder setting, and one in a day-patient setting (27). In all three studies, the anorexia nervosa treatments were CBT-based, consisting of empirically supported interventions. With regard to the nature of the offered PTSD treatments, one used Imagery Rescripting (ImRs; 29), one used Multidiagnostic Eating Disorder-Dialectical Behavior Therapy (MED-DBT; 30, 31), and one used CBT for PTSD (32) combined with Dialectical Behavior Therapy.

### Quality assessment

The included studies were all assessed as at a serious risk of bias. Due to the particularly small sample sizes, bias with regard to baseline confounding and participants selection is inevitable. None of the studies acknowledged an *a priori* protocol. As this systematic review concerns an under-researched area, no studies were excluded based on their quality rating.

See appendix B for the complete ROBINS-I assessment of the included studies.

### Anorexia nervosa – PTSD

As mentioned, the search for concurrent trauma-focused treatments offered to patients with anorexia nervosa and PTSD yielded three studies; one proof-of-concept case series study by Ten Napel-Schutz et al. (26) on ten patients, one case series study by Federici & Wisniewski (27) on seven patients from which two anorexia nervosa patients could be included, and one case report by Svaldi (28).

In the Ten Napel-Schutz study 26, findings on nine anorexia nervosa-PTSD patients and one OSFED-PTSD patient were reported, who received ImRs treatment during inpatient anorexia nervosa treatment. The inpatient CBT-based anorexia nervosa treatment focused on restoring underweight status and addressing the maintaining factors of the anorexia nervosa. The inpatient program consisted of several CBT groups, psychoeducational groups, a body- and movement-oriented therapy group, a psychotherapy group and family meetings. While being hospitalized, twelve 90-minutes individual ImRs sessions were offered twice weekly. Two participants dropped out early on; one patient with concurrent autism spectrum disorder reported “difficulties participating in the treatment”, and one patient indicated that “trauma treatment took up too much of her energy which was needed for her anorexia nervosa treatment”; these two participants were not taken along in the final reports on the ten remaining participants. At end-of-treatment, mean BMI of all participants increased, although other eating disorder pathology did not change. At end-of-treatment, a decrease or stabilization of PTSD symptoms was reported for seven participants. The authors reported that PTSD symptoms as well as eating disorder symptoms deteriorated at the start of anorexia nervosa treatment, while slowly improving after the start of trauma-focused treatment, with most improvements noted during follow-up.

With regard to feasibility, the authors reported positive effects for trauma processing, even in the early phase of the eating disorder treatment. During trauma treatment, adverse events did occur in three participants; all participants could continue both anorexia nervosa treatment as well as trauma-focused treatment despite the occurrence of adverse events. With regard to acceptability, participants reported that processing trauma interventions at the same time as receiving intensive eating disorder treatment was supportive and helped them with regaining weight (33). Next, they reported that being offered an integrated treatment gave them hope (33).

In the Federici & Wisniewski (27) study on multi-diagnostic eating disorder patients, the two anorexia nervosa-PTSD participants were offered Multidiagnostic Eating Disorder-DBT therapy (MED-DBT; 31) within an intensive, outpatient group based setting over six months. This MED-DBT program combines empirically supported CBT approaches targeted at eating disorder pathology, with standard DBT modalities and strategies targeted at trauma symptoms. The eating disorder interventions included establishing a regular eating pattern, weekly weigh-ins, self-monitoring of food and body weight, psychoeducation, teaching self-control and problem-solving strategies, managing eating disorder cognitions and stimulus control. In addition, weekly individual DBT therapy sessions were offered.

With regard to feasibility, serious adverse events were reported on one participant; because of four hospitalizations due to cardiac problems secondary to her eating disorder symptoms, one participant had to stop treatment after five weeks. The participant who completed treatment reached a normal weight, stopped vomiting and stopped excessively exercising. At end-of-treatment, a considerable decrease in self-injurious and suicidal behaviors was reported.

The 22-years old patient described in the single case report by Svaldi (28) received inpatient care for her anorexia nervosa over a period of 22 weeks. This inpatient treatment was CBT-based and consisted of group therapy sessions, creative therapy, movement therapy, weekly individual therapy sessions and supervised meals and snacks. Treatment was aimed at normalizing the eating pattern, ending laxative misuse, restoring feelings of hunger and satiety, restoring underweight with 700 grams a week, and improving poor body image and low self-esteem. The concurrently offered PTSD treatment consisted of individual 50-90 minutes CBT for PTSD combined with DBT sessions, offered twice a week during 22 weeks. With regard to feasibility, the author stated that the patient was able to process trauma-focused treatment interventions while keeping up weight regain. Near the end of treatment, a temporarily deterioration of both eating disorder as well as PTSD symptoms was reported. At end-of-treatment, anorexia nervosa pathology substantially improved and PTSD reached partial remission.

Please see **Table 1**. for all characteristics of the included studies.

**Table 1:** Characteristics of the included studies.

Study, year	Treatment methods	Design	N	Baseline weight	Sample characteristics & Sociodemographic characteristics	Outcome measures	(Serious) Adverse Events	End-of-treatment effect	
								Anorexia nervosa	Post-traumatic stress disorder
Search 1 Anorexia nervosa - Post-traumatic stress disorder									
Ten Napel et al., 2022	Inpatient CBT eating disorder treatment Imagery Rescripting 12 90- minutes sessions, twice a week	Case series study	10	BMI range 14.6 – 16.5	DSM-5 anorexia nervosa, subtype unknown (*n* = 9) DSM-5 OSFED (*n* =1) DSM-5 Post-traumatic stress disorder (*N* = 10) Age: range 16-58 years, mean = 26.4 Sex assigned at birth: female (*N* = 10) Nationality: Dutch Vocational education: *n* = 6 Senior secondary education: *n* = 4	Body mass index; Eating Disorder Examination-Questionnaire; PTSD Scale-Self Report for DSM-5; Visual Analogue Scales; Post-Traumatic Cognitions Inventory; Difficulties in Emotion Regulation Scale. Client perspectives.	In one patient conversion and psychotic symptoms worsened. In two more patients a temporarily deterioration of symptoms occurred.	Mean BMI raised from 16.55 till 17.80 Eating disorder pathology did not change significantly.	7/10 patients reported decrease or stabilization in PTSD related symptoms. Emotion regulation problems did not change significantly.
Federici & Wisniewski 2013, (27)	Day-patient - Multidiagnostic Eating Disorder- Dialectical Behavior Therapy Duration 24 weeks	Case series study	2 (of 7)	Patient 1 BMI = 16.7	Patient 1 DSM-IV-TR anorexia nervosa, binge-eating/purging subtype Post-traumatic stress disorder Borderline personality disorder Age:23 Sex assigned at birth: female Race: NR Ethnicity: White/Caucasian Socioeconomic status: NR	Body mass index; Eating Disorder Examination-Questionnaire; The Deliberate Self-Harm Inventory; Medical Stability; Client perspectives; Clinician perspectives.		Patient 1 BMI = 18.8, vomiting & excessive exercise stopped	Patient 1 Decrease self-injurious and suicidal behaviors. Also a considerable decrease in hospital admissions.
				Patient 2 BMI = 16.9	Patient 2 DSM-IV-TR anorexia nervosa, binge-eating/purging subtype Post-traumatic stress disorder Major depression disorder Obsessive compulsive disorder Borderline personality disorder traits Age: 24 Sex assigned at birth: female Race: NR Ethnicity: White/Caucasian Socioeconomic status: NR		Patient 2 stopped treatment due to 4 hospitalizations due to therapy inferring behavior.	Not applicable	Not applicable
Svaldi, J., 2004 (28)	Inpatient CBT eating disorder treatment Duration 22 weeks CBT PTSD& Dialectical Behavior Therapy, twice a week, 50-90 minutes	Case report	1	BMI = 15.4	ICD-10 anorexia nervosa, binge-eating/purging subtype ICD-10 Post-traumatic stress disorder ICD-10 Major depressive disorder ICD-10 Avoidant personality disorder Age: 22 Sex assigned at birth: female Nationality: German Socioeconomic status: NR	Body mass index; Beck Depression Inventory; Symptom-Check-List 90-Revised.	No (serious) adverse events reported. At ending treatment a temporarily deterioration of symptoms occurred.	BMI = 19.7 Laxative misuse stopped	PTSD partial in remission, avoidant behaviors with regard to worst elements trauma still present.

Of the 13 participants in the three included studies, serious adverse events were reported on one participant; she needed to be hospitalized four times during the first five weeks of treatment, due to cardiac problems secondary to her eating disorder and she was advised to stop treatment (27). Adverse events and/or a temporarily deterioration of symptoms were reported in four participants (*n* = 3, 26; *N* = 1, 28). All four participants were able to continue with the concurrent treatment despite the occurrence of adverse events.

With regard to treatment effect on anorexia nervosa, weight regain was reported on all 12 participants who completed treatment. In the two participants who completed treatment and were diagnosed with binge-eating/purging subtype, at end-of-treatment purging behaviors stopped. With regard to the effectiveness of PTSD treatment, stabilization or improvement in PTSD symptoms was reported on eight participants at end-of-treatment, however in one participant PTSD outcome was not specifically reported (27).

Due to the small study sample, no comparisons by type of trauma, by type of trauma-focused treatment or by participants characteristics (i.e. severity of anorexia nervosa pathology, presence & nature of multimorbidity) could be made.

## Discussion

This systematic review explored the feasibility, acceptability and treatment effects of psychological treatments simultaneously offered to patients with anorexia nervosa and comorbid PTSD. When the presence of comorbid PTSD hinders patients to regain weight, they are at risk of developing a chronic status of both anorexia nervosa and PTSD, if the PTSD goes untreated. Findings of this review highlight that a dearth of treatment research on anorexia nervosa and comorbid PTSD exists, as after an extensive, systematic search only three pilot studies could be included in which trauma-focused treatment was offered to underweight patients. The call for an increase in treatment research on comorbidity in anorexia nervosa, as was done over two decades ago by leading experts in the field, has not led to a significant growth.

The three included observational studies were all assessed as having a serious risk of bias (26–28). Two studies took place in Europa (Germany and the Netherlands) and one study took place in the USA. The included studies reported on a total of 13 female participants, between 16 and 58 years old with a baseline BMI ranging between 14.6 and 16.5. Of the 13 participants included in this review who started simultaneous treatments, 12 participants were able to complete both the anorexia nervosa and the trauma-focused treatment.

In all three studies, the anorexia treatment was intensive, with a minimum duration of three months. Next, all three eating disorder treatments were CBT-based and consisted of empirically supported interventions. Weight regain was reported for all 12 treatment completers, purging behaviors stopped in the two participants who were diagnosed with binge-eating/purging subtype and who completed treatment.

With regard to the concurrently offered PTSD treatment, end-of-treatment stabilization or improvement in PTSD symptoms was reported in eight participants, in one participant PTSD outcome was not specifically reported, besides a decrease in self-injurious behaviors, in suicidal behaviors and a decrease in hospital admissions. (27).

The reported dropout rates in the included studies may be regarded as relatively optimistic, as usually dropout rates are typically high in anorexia nervosa treatment, particularly when patients have a comorbid PTSD (3). One might preliminarily hypothesize that the attention being given to PTSD symptomatology may help anorexia nervosa patients staying engaged in the offered eating disorder treatment, as is suggested elsewhere (18). This beneficial effect of offering trauma-focused treatment to patients with a history of unsuccessful, unsuitable treatments is described by the participants in the Ten Napel-Schutz study (33) as they reported that being offered a concurrent treatment gave them hope, an essential ingredient for motivation.

Regarding the feasibility of concurrent treatment, the emotional and cognitive functioning of all participants in the included studies was sufficient to process trauma-focused interventions despite their serious underweight status.

As there are no comparison groups in the included studies, the frequency of (serious) adverse events occurring during these concurrent treatments cannot be compared to the frequency of (serious) adverse events occurring in regular anorexia nervosa treatments with similar study populations.

With regard to PTSD outcome, as all participants were receiving intensive eating disorder treatment during trauma-focused treatment, specific effects of PTSD treatment are difficult to isolate.

### Strengths and limitations

To our knowledge, this review is the first to systematically examine feasibility, acceptability and treatment effects of concurrent PTSD treatment approaches offered to patients with anorexia nervosa or OSFED with moderate, severe or extreme baseline underweight status. Furthermore, the search was extensive and systematic with broad search terms and with few restrictions set. Due to the nature of the included studies, limitations are inevitable. Given all studies had an observational study design and reported on very small sample sizes, they were assessed as low-quality. At the same time, demonstrating initial implementation of experimental interventions, usually takes place under uncontrolled conditions in pilot studies with small sample sizes. To our knowledge, these are the only studies worldwide in which anorexia nervosa patients were actually offered trauma-focused interventions while being in an significant underweight status to date.

### Clinical implications

Innovative treatment research on concurrent treatment methods is, in part, hindered by refraining anorexia nervosa patients with low body weight from trauma-focused clinical trials due to fear of symptom exacerbation, fear of relapse and due to the assumption that psychological interventions cannot be processed when patients are underweight. The severely underweight patients who participated in the studies included in this review were able to emotionally and cognitively process the trauma-focused interventions offered.

Refraining patients with anorexia nervosa from trauma-focused treatments may not be warranted and perspectives for larger, high-quality clinical studies on concurrent treatment approaches are opened.

## Author contributions

EvdB: Conceptualization, Investigation, Methodology, Validation, Writing – original draft, Writing – review & editing. KP: Conceptualization, Data curation, Investigation, Methodology, Project administration, Software, Writing – review & editing, Writing – original draft. CP: Data curation, Methodology, Software, Writing – review & editing, Writing – original draft. PD: Conceptualization, Methodology, Writing – review & editing, Writing – original draft. EvB: Conceptualization, Methodology, Validation, Writing – review & editing, Writing – original draft. MJ: Methodology, Writing – original draft, Writing – review & editing, Supervision. CC: Methodology, Writing – original draft, Writing – review & editing, Conceptualization, Validation. JD: Supervision, Writing – original draft, Writing – review & editing.

## References

[1] Eddy KT Tabri N Thomas JJ Murray HB Keshaviah A Hastings E. Recovery from anorexia nervosa and bulimia nervosa at 22-year follow-up. J Clin Psychiatry (2017) 78:184–9. doi: 10.4088/JCP.15m10393 PMC7883487 28002660

[2] Keski-Rahkonen A Mustelin L. Epidemiology of eating disorders in Europe: prevalence, incidence, comorbidity, course, consequences, and risk factors. Curr Opin Psychiatry (2016) 29:340–5. doi: 10.1097/YCO.0000000000000278 27662598

[3] Day S Hay P Tannous WK Fatt SJ Mitchison D. A systematic review of the effect of PTSD and trauma on treatment outcomes for eating disorders. Trauma Violence Abuse (2023). doi: 10.1177/15248380231167399 PMC10913314 37125723

[4] Caslini M Bartoli F Crocamo C Dakanalis A Clerici M Carrà G. Disentangling the association between child abuse and eating disorders: a systematic review and meta-analysis. Psychosomatic Med (2016) 78:79–90. doi: 10.1097/PSY.0000000000000233 26461853

[5] Molendijk ML Hoek HW Brewerton TD Elzinga BM. Childhood maltreatment and eating disorder pathology: a systematic review and dose-response meta-analysis. psychol Med (2017) 47(8):1–15. doi: 10.1017/S0033291716003561 28100288

[6] Brewerton TD Brady K. The role of stress, trauma, and PTSD in the etiology and treatment of eating disorders, addictions, and substance use disorders. In: Brewerton TD Dennis AB, editors. Eating disorders, addictions and substance use disorders: Research, clinical and treatment perspectives Berlin, Heidelberg: Springer- Verlag Publishing/Springer Nature (2014). p. 379–404. doi: 10.1007/978-3-642-45378-6_17

[7] Oprel D Hoeboer C Schoorl M De Kleine R Cloitre M Wigard I. Effect of Prolonged Exposure, intensified Prolonged Exposure and STAIR+Prolonged Exposure in patients with PTSD related to childhood abuse: a randomized controlled trial. Eur J Psychotraumatol (2021) 12:1. doi: 10.1080/20008198.2020.1851511 34630934 PMC8500700

[8] Tagay S Schlottbohm E Reyes-Rodriguez ML Repic N Senf W. Eating disorders, trauma, PTSD, and psychosocial resources. Eating Disord (2014) 22:33–49. doi: 10.1080/10640266.2014.857517 PMC3966425 24365526

[9] Schoemaker C Smit F Bijl RV Vollebergh WAM. Bulimia nervosa following psychological and multiple child abuse: Support for the self-medication hypothesis in a population-based cohort study. Int J eating Disord (2002) 32:381–8. doi: 10.1002/eat.10102 12386903

[10] Trottier K Monson CM Wonderlich SA Olmsted MP. Initial Findings From Project Recover: Overcoming Co-Occurring Eating Disorders and Posttraumatic Stress Disorder Through Integrated Treatment. J Trauma Stress, (2017) 30(2):173–177. doi: 10.1002/jts.22176 28398626

[11] Rijkers C Schoorl M Van Hoeken D Hoek HW. Eating disorders and posttraumatic stress disorder. Curr Opin Psychiatry (2019) 32:510–7. doi: 10.1097/YCO.0000000000000545 31313708

[12] Cassioli E Rossi E D’Anna G Martelli M Hazzard VM Crosby RD. A 1-year follow-up study of the longitudinal interplay between emotion dysregulation and childhood trauma in the treatment of anorexia nervosa. Int J Eating Disord (2021) 55:98–107. doi: 10.1002/eat.23647 PMC8787461 34862809

[13] Convertino AD Mendoza RR. Posttraumatic stress disorder, traumatic events, and longitudinal eating disorder treatment outcomes: A systematic review. Int J Eating Disord (2023) 56:1055–74. doi: 10.1002/eat.23933 PMC10247514 36916450

[14] NICE National Institute for Health and Care Excellence. Eating disorders: Recognition and treatment. Version 2.0 National Institute for Health and Care Excellence (2017)

[15] APA. (2017). *Clinical Practice Guideline for the Treatment of Posttraumatic Stress Disorder (PTSD) in Adults* . In: Clinical Practice Guideline for the Treatment of Posttraumatic Stress Disorder (PTSD) in Adults (apa.org) Washington DC: American Psychological Association (2023).

[16] Crone C Fochtmann LJ Attia E Boland R Escobar J Fornari V. The american psychiatric association practice guideline for the treatment of patients with eating disorders. Am J Psychiatry (2023) 180:167–171. doi: 10.1176/appi.ajp.23180001 36722117

[17] Ronconi JM Shiner B Watts BV. Inclusion and exclusion criteria in randomized controlled trials of psychotherapy for PTSD. J Psychiatr Pract (2014) 20:25–37. doi: 10.1097/01.pra.0000442936.23457.5 24419308

[18] Brewerton TD. The integrated treatment of eating disorders, posttraumatic stress disorder, and psychiatric comorbidity: a commentary on the evolution of principles and guidelines. Front Psychiatry (2023) 14:1149433. doi: 10.3389/fpsyt.2023.1149433 37252137 PMC10213703

[19] Van Elburg A Danner U Sternheim L Lammers M Elzakkers I. Mental capacity, decision-making and emotion dysregulation in severe enduring anorexia nervosa, (2021). Front Psychiatry (2021) 12. doi: 10.3389/fpsyt.545317 PMC7991306 33776810

[20] Obeid N McVey G Seale E Preskow W Norris ML. Cocreating research priorities for anorexia nervosa: The Canadian Eating Disorder Priority Setting Partnership. Int J Eating Disord (2020) 53:392–402. doi: 10.1002/eat.23234 32011022

[21] Agras WS Brandt HA Bulik CM Dolan-Sewell R Fairburn CG Halmi KA. Report of the National Institutes of Health workshop on overcoming barriers to treatment research in anorexia nervosa. Int J Eating Disord (2004) 35:509–21. doi: 10.1002/eat.10261 15101067

[22] Trottier K Monson C Wonderlich S Crosby R. Results of the first randomized controlled trial of integrated cognitive-behavioral therapy for eating disorders and posttraumatic stress disorder. psychol Med (2022) 52:587–96. doi: 10.1017/S0033291721004967 PMC8883823 34872625

[23] Claudat K Reilly EE Convertino AD Trim J Cusack A Kaye WH. Integrating evidence-based PTSD treatment into intensive eating disorders treatment: a preliminary investigation. Eating Weight Disord -Studies Anorexia Bulimia Obes (2022) 27:3599–607. doi: 10.1007/s40519-022-01500-9 PMC9803734 36401788

[24] Page MJ McKenzie JE Bossuyt PM Boutron I Hoffmann TC Mulrow CD. The PRISMA 2020 statement: an updated guideline for reporting systematic reviews. BMJ (2021) 372:n71. doi: 10.1136/bmj.n71 33782057 PMC8005924

[25] Sterne JA Hernán MA Reeves BC Savović J Berkman ND Viswanathan M. ROBINS-I: a tool for assessing risk of bias in non-randomised studies of interventions. BMJ (Clinical Res ed.) (2016) 355:i4919. doi: 10.1136/bmj.i4919 PMC5062054 27733354

[26] Ten Napel-Schutz MC Vroling M Mares SHW Arntz A. Treating PTSD with Imagery Rescripting in underweight eating disorder patients: a multiple baseline case series study. J Eating Disord (2022) 10:35. doi: 10.1186/s40337-022-00558-1 PMC8908690 35264254

[27] Federici A Wisniewski L. An intensive DBT program for patients with multidiagnostic eating disorder presentations: A case series analysis. Int J Eating Disord (2013) 46:322–31. doi: 10.1002/eat.22112 23381784

[28] Svaldi JJ. Kognitiv-verhaltenstherapeutische Behandlung einer Patientin mit Posttraumatischer Belastungsstörung und Anorexia nervosa. Verhaltenstherapie Verhaltensmedizin (2004) 25:231–50.

[29] Arntz A. Imagery rescripting as a therapeutic technique: Review of clinical trials, basic studies, and research agenda. J Exp Psychopathol (2012) 3:189–208. doi: 10.5127/jep.024211

[30] Linehan MM. Trainingsmanual zur dialektisch-behavioralen Therapie der Borderline Persönlichkeitsstöring Göttingen: CIP Medien (1996).

[31] Federici A Wisniewski L Ben-Porath D. Description of an intensive dialectical behavior therapy program for multidiagnostic clients with eating disorders. J Couns Dev (2012) 90:330–8. doi: 10.1002/j.1556-6676.2012.00041.x

[32] Ehlers A. Posttraumatische Belastungsstörung Göttingen: Hogrefe (1999).

[33] Ten Napel-Schutz MC Karbouniaris S Mares SHW Arntz A Abma TA. Perspectives of underweight people with eating disorders on receiving Imagery Rescripting trauma treatment: a qualitative study of their experiences. J Eating Disord (2022) 10:188. doi: 10.1186/s40337-022-00712-9 PMC9710063 36451217

