# Supplementary material for: Treatment of patients with anorexia nervosa and comorbid post-traumatic stress disorder; where do we stand? A systematic scoping review
Source: Front Psychiatry. 2024 Feb 26;15:1365715. doi: 10.3389/fpsyt.2024.1365715 (PMC10925640; doi:10.3389/fpsyt.2024.1365715)
Supplement: Supplementary file 1 [file DataSheet_1.docx]

| **Database searched** | **Platform** | **Years of coverage** | **19-6-2023** | **Deduplicated** |
| --- | --- | --- | --- | --- |
| PubMed | PubMed | 1946 – Present | 286 | 286 |
| Embase | Embase.com | 1971 - Present | 1.256 | 1.028 |
| APA PsycInfo | EBSCO | 1968 - Present | 211 | 62 |
| Web of Science | Clarivate | 1975 - Present | 457 | 209 |
| Scopus | Elsevier | 1970 - Present | 988 | 161 |
| Cochrane CENTRAL | Wiley | 1992 - Present | 55 | 23 |
| **Total:** |  |  | **3.253** | **1.769** |

**Pubmed**

| Search | Query | Results |
| --- | --- | --- |
| #4 | Search: #1 AND #2 AND #4 Sort by: Most Recent | [286](https://pubmed.ncbi.nlm.nih.gov/?term=%231+AND+%232+AND+%234&sort=date) |
| #3 | Search: "therapy" [Subheading] OR "Psychotherapy"[Mesh] OR "Psychoanalysis"[Mesh] OR "Eye Movement Desensitization Reprocessing"[Mesh] OR "Telemedicine"[Mesh] OR "Mobile Applications"[Mesh] OR "Social Media"[Mesh] OR "Therapy, Computer-Assisted"[Mesh:NoExp] OR "Telecommunications"[Mesh:NoExp] OR "Electronic Mail"[Mesh] OR "Videoconferencing"[Mesh] OR "Cell Phone"[Mesh] OR "Distance Counseling"[Mesh] OR "Wearable Electronic Devices"[Mesh] OR "Counseling"[Mesh] OR Psychoanalys*[tiab] OR treatment*[tiab] OR psychotherap*[tiab] OR therap*[tiab] OR "Eye Movement Desensitization Reprocessing"[tiab] OR EMDR[tiab] OR intervent*[tiab] OR imagery[tiab] OR "cognitive behavio*"[tiab] OR "cognitive remediation*"[tiab] OR CBT[tiab] OR "mindfulness*"[tiab] OR "self-compassion*"[tiab] OR "schema focused"[tiab] OR "trauma focused"[tiab] OR mantra[tiab] OR "maudsley model"[tiab] OR "CBT-E"[tiab] OR "enhanced cogniti*"[tiab] OR "Kognitive Verhaltenstherap*"[tiab] OR KGT[tiab] OR exposur*[tiab] OR telehealth[tiab] OR "tele-health"[tiab] OR telepsychology[tiab] OR "tele-psychology"[tiab] OR telepsychiatry[tiab] OR "tele-psychiatry"[tiab] OR "tele-therap*"[tiab] OR teletherap*[tiab] OR "tele-medicine"[tiab] OR telemedicine[tiab] OR telecare[tiab] OR "tele-care"[tiab] OR telecommunicat*[tiab] OR "tele-communicat*"[tiab] OR teleconference*[tiab] OR "tele-conferenc*"[tiab] OR videoconferenc*[tiab] OR "video-conferenc*"[tiab] OR computer*[tiab] OR electronic*[tiab] OR digital*[tiab] OR ehealth[tiab] OR "e-health"[tiab] OR "e-treat*"[tiab] OR "e-therap*"[tiab] OR mhealth[tiab] OR "m-health"[tiab] OR "internet-based*"[tiab] OR "internet treat*"[tiab] OR "internet intervention*"[tiab] OR "internet counsel*"[tiab] OR "distance counsel*"[tiab] OR "web-based*"[tiab] OR cybercounsel*[tiab] OR "cyber-counsel*"[tiab] OR "online treat*"[tiab] OR "online therap*"[tiab] OR "online intervention*"[tiab] OR "online prevention*"[tiab] OR "online counsel*"[tiab] OR "text-messag*"[tiab] OR textmessag*[tiab] OR SMS[tiab] OR texting*[tiab] OR "short message service*"[tiab] OR mobile*[tiab] OR smartphone*[tiab] OR "cell-phone*"[tiab] OR cellphone*[tiab] OR "cellular phone*"[tiab] OR blended*[tiab] OR "software app*"[tiab] OR "handheld device*"[tiab] OR "hand held device*"[tiab] OR iPad*[tiab] OR iPhone*[tiab] OR email*[tiab] OR "e-mail*"[tiab] OR sensor*[tiab] OR wearable*[tiab] OR "social media*"[tiab] OR "social network*"[tiab] OR "e-counsel*"[tiab] OR ecounsel*[tiab] OR palmtop*[tiab] OR telephone*[tiab] OR WhatsApp[tiab] OR Twitter[tiab] OR Facebook[tiab] OR Instagram[tiab] OR forum[tiab] OR chat*[tiab] OR "virtual reality*"[tiab] OR avatar*[tiab] OR "Conversational agent*"[tiab] OR "virtual coach"[tiab] OR "virtual agent*"[tiab] OR "embodied agent*"[tiab] OR "relational agent*"[tiab] OR "interactive agent*"[tiab] OR "virtual character*"[tiab] OR "virtual human*"[tiab] OR "virtual assistant*"[tiab] OR VR[tiab] OR "serious game*"[tiab] OR "serious gaming"[tiab] OR gamification[tiab] OR "counseling"[tiab] OR "counselling"[tiab] Sort by: Most Recent | [14,131,104](https://pubmed.ncbi.nlm.nih.gov/?term=longqueryd992d01d42aacb3401d4&sort=date) |
| #2 | Search: "Stress Disorders, Traumatic"[Mesh] OR "Psychological Distress"[Mesh] OR "complex trauma*"[tiab] OR "posttraumatic stress*"[tiab] OR "post-traumatic stress*"[tiab] OR "posttraumatic-disorder*"[tiab] OR "post-traumatic disorder*"[tiab] OR "post traumatic neuros*"[tiab] OR "posttraumatic neuros*"[tiab] OR "traumatic distress*"[tiab] OR "posttraumatic psychos*"[tiab] OR "post-traumatic psychos*"[tiab] OR "posttraumatic syndrom*"[tiab] OR "post-traumatic syndrom*"[tiab] OR "trauma-and-stressor-related-disorder*"[tiab] OR "traumatic stress disorder*"[tiab] OR ptsd*[tiab] OR ptss*[tiab] OR "combat-disorder*"[tiab] OR "combat-fatigue*"[tiab] OR "combat-stress*"[tiab] OR "shell-shock*"[tiab] OR "combat-neuros*"[tiab] OR "war-neuros*"[tiab] OR "battle-fatigue*"[tiab] OR "psychological stress*"[tiab] OR "psychological trauma*"[tiab] OR psychotrauma*[tiab] OR "psycho-trauma*"[tiab] OR "trauma-focused*"[tiab] OR "psychological distress*"[tiab] OR "emotional trauma*"[tiab] OR "emotional distress*"[tiab] OR "emotional damag*"[tiab] OR "emotional injur*"[tiab] OR "mental damag*"[tiab] OR "mental harm*"[tiab] OR "mental injur*"[tiab] OR "mental trauma*"[tiab] OR "sexual trauma*"[tiab] OR "psychological damag*"[tiab] OR "psychological harm*"[tiab] OR "psychological injur*"[tiab] OR "Posttraumatische Belastungsstörung"[tiab] OR PTBS[tiab] Sort by: Most Recent | [116,833](https://pubmed.ncbi.nlm.nih.gov/?term=longquery0f9a27c35619d5b72043&sort=date) |
| #1 | Search: "Anorexia"[Mesh] OR "Anorexia Nervosa"[Mesh] OR "anorexi*"[tiab] OR "anorectic"[tiab] OR "underweight*"[tiab] Sort by: Most Recent | [58,821](https://pubmed.ncbi.nlm.nih.gov/?term=%22Anorexia%22%5BMesh%5D+OR+%22Anorexia+Nervosa%22%5BMesh%5D+OR+%E2%80%9Canorexi%2A%E2%80%9D%5Btiab%5D+OR+%E2%80%9Canorectic%E2%80%9D%5Btiab%5D+OR+%E2%80%9Cunderweight%2A%E2%80%9D%5Btiab%5D+&sort=date) |

Key: [Mesh]= medical subject heading, [tiab] = title, abstract, author supplied keywords.

**Embase**

| **No.** | **Query** | **Results** |
| --- | --- | --- |
| **#4** | **#1** AND **#2** AND **#3** | **1,256** |
| **#3** | **'therapy'**/lnk OR **'psychotherapy'**/exp OR **'psychoanalysis'**/exp OR **'mobile application'**/exp OR **'social media'**/exp OR **'computer assisted therapy'**/de OR **'telecommunication'**/exp OR **'e-mail'**/exp OR **'videoconferencing'**/exp OR **'mobile phone'**/exp OR **'wearable computer'**/exp OR **'counseling'**/exp OR **treatment***:ab,ti,kw OR **psychotherap***:ab,ti,kw OR **therap***:ab,ti,kw OR **'eye movement desensitization reprocessing'**:ab,ti,kw OR **emdr**:ab,ti,kw OR **intervent***:ab,ti,kw OR **imagery**:ab,ti,kw OR **'cognitive behavio*'**:ab,ti,kw OR **'cognitive remediation*'**:ab,ti,kw OR **cbt**:ab,ti,kw OR **'mindfulness*'**:ab,ti,kw OR **'self-compassion*'**:ab,ti,kw OR **'schema focused'**:ab,ti,kw OR **'trauma focused'**:ab,ti,kw OR **mantra**:ab,ti,kw OR **'maudsley model'**:ab,ti,kw OR **'cbt-e'**:ab,ti,kw OR **'enhanced cogniti*'**:ab,ti,kw OR **'kognitive verhaltenstherap*'**:ab,ti,kw OR **kgt**:ab,ti,kw OR **exposur***:ab,ti,kw OR **telehealth**:ab,ti,kw OR **'tele-health'**:ab,ti,kw OR **telepsychology**:ab,ti,kw OR **'tele-psychology'**:ab,ti,kw OR **telepsychiatry**:ab,ti,kw OR **'tele-psychiatry'**:ab,ti,kw OR **'tele-therap*'**:ab,ti,kw OR **teletherap***:ab,ti,kw OR **'tele-medicine'**:ab,ti,kw OR **telemedicine**:ab,ti,kw OR **telecare**:ab,ti,kw OR **'tele-care'**:ab,ti,kw OR **telecommunicat***:ab,ti,kw OR **'tele-communicat*'**:ab,ti,kw OR **teleconference***:ab,ti,kw OR **'tele-conferenc*'**:ab,ti,kw OR **videoconferenc***:ab,ti,kw OR **'video-conferenc*'**:ab,ti,kw OR **computer***:ab,ti,kw OR **electronic***:ab,ti,kw OR **digital***:ab,ti,kw OR **ehealth**:ab,ti,kw OR **'e-health'**:ab,ti,kw OR **'e-treat*'**:ab,ti,kw OR **'e-therap*'**:ab,ti,kw OR **mhealth**:ab,ti,kw OR **'m-health'**:ab,ti,kw OR **'internet-based*'**:ab,ti,kw OR **'internet treat*'**:ab,ti,kw OR **'internet intervention*'**:ab,ti,kw OR **'internet counsel*'**:ab,ti,kw OR **'distance counsel*'**:ab,ti,kw OR **'web-based*'**:ab,ti,kw OR **cybercounsel***:ab,ti,kw OR **'cyber-counsel*'**:ab,ti,kw OR **'online treat*'**:ab,ti,kw OR **'online therap*'**:ab,ti,kw OR **'online intervention*'**:ab,ti,kw OR **'online prevention*'**:ab,ti,kw OR **'online counsel*'**:ab,ti,kw OR **'text-messag*'**:ab,ti,kw OR **textmessag***:ab,ti,kw OR **sms**:ab,ti,kw OR **texting***:ab,ti,kw OR **'short message service*'**:ab,ti,kw OR **mobile***:ab,ti,kw OR **smartphone***:ab,ti,kw OR **'cell-phone*'**:ab,ti,kw OR **cellphone***:ab,ti,kw OR **'cellular phone*'**:ab,ti,kw OR **blended***:ab,ti,kw OR **'software app*'**:ab,ti,kw OR **'handheld device*'**:ab,ti,kw OR **'hand held device*'**:ab,ti,kw OR **ipad***:ab,ti,kw OR **iphone***:ab,ti,kw OR **email***:ab,ti,kw OR **'e-mail*'**:ab,ti,kw OR **sensor***:ab,ti,kw OR **wearable***:ab,ti,kw OR **'social media*'**:ab,ti,kw OR **'social network*'**:ab,ti,kw OR **'e-counsel*'**:ab,ti,kw OR **ecounsel***:ab,ti,kw OR **palmtop***:ab,ti,kw OR **telephone***:ab,ti,kw OR **whatsapp**:ab,ti,kw OR **twitter**:ab,ti,kw OR **facebook**:ab,ti,kw OR **instagram**:ab,ti,kw OR **forum**:ab,ti,kw OR **chat***:ab,ti,kw OR **'virtual reality*'**:ab,ti,kw OR **avatar***:ab,ti,kw OR **'conversational agent*'**:ab,ti,kw OR **'virtual coach'**:ab,ti,kw OR **'virtual agent*'**:ab,ti,kw OR **'embodied agent*'**:ab,ti,kw OR **'relational agent*'**:ab,ti,kw OR **'interactive agent*'**:ab,ti,kw OR **'virtual character*'**:ab,ti,kw OR **'virtual human*'**:ab,ti,kw OR **'virtual assistant*'**:ab,ti,kw OR **vr**:ab,ti,kw OR **'serious game*'**:ab,ti,kw OR **'serious gaming'**:ab,ti,kw OR **gamification**:ab,ti,kw OR **'counseling'**:ab,ti,kw OR **'counselling'**:ab,ti,kw | **14,717,493** |
| **#2** | **'posttraumatic stress disorder'**/exp OR **'distress syndrome'**/exp OR **'complex trauma*'**:ab,ti,kw OR **'posttraumatic stress*'**:ab,ti,kw OR **'post-traumatic stress*'**:ab,ti,kw OR **'posttraumatic-disorder*'**:ab,ti,kw OR **'post-traumatic disorder*'**:ab,ti,kw OR **'post traumatic neuros*'**:ab,ti,kw OR **'posttraumatic neuros*'**:ab,ti,kw OR **'traumatic distress*'**:ab,ti,kw OR **'posttraumatic psychos*'**:ab,ti,kw OR **'post-traumatic psychos*'**:ab,ti,kw OR **'posttraumatic syndrom*'**:ab,ti,kw OR **'post-traumatic syndrom*'**:ab,ti,kw OR **'trauma-and-stressor-related-disorder*'**:ab,ti,kw OR **'traumatic stress disorder*'**:ab,ti,kw OR **ptsd***:ab,ti,kw OR **ptss***:ab,ti,kw OR **'combat-disorder*'**:ab,ti,kw OR **'combat-fatigue*'**:ab,ti,kw OR **'combat-stress*'**:ab,ti,kw OR **'shell-shock*'**:ab,ti,kw OR **'combat-neuros*'**:ab,ti,kw OR **'war-neuros*'**:ab,ti,kw OR **'battle-fatigue*'**:ab,ti,kw OR **'psychological stress*'**:ab,ti,kw OR **'psychological trauma*'**:ab,ti,kw OR **psychotrauma***:ab,ti,kw OR **'psycho-trauma*'**:ab,ti,kw OR **'trauma-focused*'**:ab,ti,kw OR **'psychological distress*'**:ab,ti,kw OR **'emotional trauma*'**:ab,ti,kw OR **'emotional distress*'**:ab,ti,kw OR **'emotional damag*'**:ab,ti,kw OR **'emotional injur*'**:ab,ti,kw OR **'mental damag*'**:ab,ti,kw OR **'mental harm*'**:ab,ti,kw OR **'mental injur*'**:ab,ti,kw OR **'mental trauma*'**:ab,ti,kw OR **'sexual trauma*'**:ab,ti,kw OR **'psychological damag*'**:ab,ti,kw OR **'psychological harm*'**:ab,ti,kw OR **'psychological injur*'**:ab,ti,kw OR **'posttraumatische belastungsstörung'**:ab,ti,kw OR **ptbs**:ab,ti,kw | **193,997** |
| **#1** | **'anorexia'**/exp OR **'anorexia nervosa'**/exp OR **'anorexi*'**:ab,ti,kw OR **'anorectic'**:ab,ti,kw OR **'underweight*'**:ab,ti,kw | **133,733** |

Key: Ab, ti, kw searches in abstract, title and author supplied keywords, /exp searches Emtree preferred indexing term, it=publication type

**PsycInfo**

| **#** | **Query** | **Limiters/Expanders** | **Results** |
| --- | --- | --- | --- |
| S5 | S4 | Limiters –  Academic journals | 211 |
| S4 | S1 AND S2 AND S3 | Search modes - Boolean/Phrase | 260 |
| S3 | DE "Psychotherapy" OR DE "Adlerian Psychotherapy" OR DE "Adolescent Psychotherapy" OR DE "Affirmative Therapy" OR DE "Analytical Psychotherapy" OR DE "Autogenic Training" OR DE "Brief Psychotherapy" OR DE "Brief Relational Therapy" OR DE "Child Psychotherapy" OR DE "Client Centered Therapy" OR DE "Conversion Therapy" OR DE "Couples Therapy" OR DE "Eclectic Psychotherapy" OR DE "Emotion Focused Therapy" OR DE "Existential Therapy" OR DE "Experiential Psychotherapy" OR DE "Expressive Psychotherapy" OR DE "Eye Movement Desensitization Therapy" OR DE "Feminist Therapy" OR DE "Geriatric Psychotherapy" OR DE "Gestalt Therapy" OR DE "Group Psychotherapy" OR DE "Guided Imagery" OR DE "Humanistic Psychotherapy" OR DE "Hypnotherapy" OR DE "Individual Psychotherapy" OR DE "Insight Therapy" OR DE "Integrative Psychotherapy" OR DE "Interpersonal Psychotherapy" OR DE "Logotherapy" OR DE "Narrative Therapy" OR DE "Network Therapy" OR DE "Persuasion Therapy" OR DE "Primal Therapy" OR DE "Psychoanalysis" OR DE "Psychodrama" OR DE "Psychodynamic Psychotherapy" OR DE "Psychotherapeutic Counseling" OR DE "Psychotherapeutic Techniques" OR DE "Rational Emotive Behavior Therapy" OR DE "Reality Therapy" OR DE "Relationship Therapy" OR DE "Solution Focused Therapy" OR DE "Strategic Therapy" OR DE "Supportive Psychotherapy" OR DE "Transactional Analysis" OR DE "Adolescent Psychotherapy" OR DE "Multisystemic Therapy" OR DE "Child Psychotherapy" OR DE "Play Therapy" OR DE "Gestalt Therapy" OR DE "Empty Chair Technique" OR DE "Group Psychotherapy" OR DE "Encounter Group Therapy" OR DE "Therapeutic Community" OR DE "Humanistic Psychotherapy" OR DE "Client Centered Therapy" OR DE "Hypnotherapy" OR DE "Age Regression (Hypnotic)" OR DE "Ericksonian Psychotherapy" OR DE "Posthypnotic Suggestions" OR DE "Integrative Psychotherapy" OR DE "Schema Therapy" OR DE "Psychoanalysis" OR DE "Adlerian Psychotherapy" OR DE "Brief Relational Therapy" OR DE "Dream Analysis" OR DE "Self-Analysis" OR DE "Psychotherapeutic Counseling" OR DE "Family Therapy" OR DE "Psychotherapeutic Techniques" OR DE "Active Listening" OR DE "Animal Assisted Therapy" OR DE "Autogenic Training" OR DE "Brief Relational Therapy" OR DE "Centering" OR DE "Cotherapy" OR DE "Dream Analysis" OR DE "Empty Chair Technique" OR DE "Ericksonian Psychotherapy" OR DE "Free Association" OR DE "Guided Imagery" OR DE "Life Review" OR DE "Mirroring" OR DE "Morita Therapy" OR DE "Motivational Interviewing" OR DE "Mutual Storytelling Technique" OR DE "Network Therapy" OR DE "Paradoxical Techniques" OR DE "Psychodrama" OR DE "Strategic Therapy" OR DE "Strategic Family Therapy" OR DE "Cognitive Therapy" OR DE "Online Therapy" OR DE "Telepsychology" OR DE "Counseling" OR DE "Treatment" OR DE "Psychotherapeutic Counseling" OR DE "Family Therapy" OR DE "Teleconferencing" OR DE "Videoconferencing" OR DE "Teleconsultation" OR DE "Telepsychiatry" OR DE "Telerehabilitation" OR DE "Digital Interventions" OR DE "Computer Assisted Therapy" OR DE "Mobile Applications" OR DE "Virtual Reality" OR DE "Augmented Reality" OR DE "Avatars" OR DE "Computer Applications" OR DE "Cloud Computing" OR DE "Computer Assisted Design" OR DE "Computer Assisted Diagnosis" OR DE "Computer Assisted Instruction" OR DE "Computer Assisted Therapy" OR DE "Computer Programming" OR DE "Computer Simulation" OR DE "Computer Software" OR DE "Electronic Collaboration" OR DE "Electronic Learning" OR DE "Groupware" OR DE "Hypermedia" OR DE "Hypertext" OR DE "Mobile Applications" OR DE "Telecommunications Media" OR DE "Radio" OR DE "Telephone Systems" OR DE "Television" OR DE "Wireless Technologies" OR DE "Social Media" OR DE "Online Social Networks" OR DE "Online Community" OR DE "Tablet Computers" OR DE "Mobile Health" OR DE "Wearable Devices" OR DE "Mobile Phones" OR DE "Mobile Devices" OR DE "Smartphones" OR DE "Text Messaging" OR TI(Psychoanalys* OR treatment* OR psychotherap* OR therap* OR “Eye Movement Desensitization Reprocessing” OR EMDR OR intervent* OR imagery OR "cognitive behavio*" OR “cognitive remediation*” OR CBT OR “mindfulness*” OR “self-compassion*” OR “schema focused” OR “trauma focused” OR mantra OR “maudsley model” OR “CBT-E” OR “enhanced cogniti*” OR “Kognitive Verhaltenstherap*” OR KGT OR exposur* OR telehealth OR “tele-health” OR telepsychology OR “tele-psychology” OR telepsychiatry OR “tele-psychiatry” OR “tele-therap*” OR teletherap* OR “tele-medicine” OR telemedicine OR telecare OR “tele-care” OR telecommunicat* OR “tele-communicat*” OR teleconference* OR “tele-conferenc*” OR videoconferenc* OR “video-conferenc*” OR computer* OR electronic* OR digital* OR ehealth OR “e-health” OR “e-treat*” OR “e-therap*” OR mhealth OR “m-health” OR “internet-based*” OR “internet treat*” OR “internet intervention*” OR “internet counsel*” OR “distance counsel*” OR “web-based*” OR cybercounsel* OR “cyber-counsel*” OR “online treat*” OR “online therap*” OR “online intervention*” OR “online prevention*” OR “online counsel*” OR “text-messag*” OR textmessag* OR SMS OR texting* OR “short message service*” OR mobile* OR smartphone* OR “cell-phone*” OR cellphone* OR “cellular phone*” OR blended* OR “software app*” OR “handheld device*” OR “hand held device*” OR iPad* OR iPhone* OR email* OR “e-mail*” OR sensor* OR wearable* OR “social media*” OR “social network*” OR “e-counsel*” OR ecounsel* OR palmtop* OR telephone* OR WhatsApp OR Twitter OR Facebook OR Instagram OR forum OR chat* OR “virtual reality*” OR avatar* OR “Conversational agent*” OR “virtual coach” OR “virtual agent*” OR “embodied agent*” OR “relational agent*” OR “interactive agent*” OR “virtual character*” OR “virtual human*” OR “virtual assistant*” OR VR OR “serious game*” OR “serious gaming” OR gamification OR “counseling” OR “counselling”) OR AB(Psychoanalys* OR treatment* OR psychotherap* OR therap* OR “Eye Movement Desensitization Reprocessing” OR EMDR OR intervent* OR imagery OR "cognitive behavio*" OR “cognitive remediation*” OR CBT OR “mindfulness*” OR “self-compassion*” OR “schema focused” OR “trauma focused” OR mantra OR “maudsley model” OR “CBT-E” OR “enhanced cogniti*” OR “Kognitive Verhaltenstherap*” OR KGT OR exposur* OR telehealth OR “tele-health” OR telepsychology OR “tele-psychology” OR telepsychiatry OR “tele-psychiatry” OR “tele-therap*” OR teletherap* OR “tele-medicine” OR telemedicine OR telecare OR “tele-care” OR telecommunicat* OR “tele-communicat*” OR teleconference* OR “tele-conferenc*” OR videoconferenc* OR “video-conferenc*” OR computer* OR electronic* OR digital* OR ehealth OR “e-health” OR “e-treat*” OR “e-therap*” OR mhealth OR “m-health” OR “internet-based*” OR “internet treat*” OR “internet intervention*” OR “internet counsel*” OR “distance counsel*” OR “web-based*” OR cybercounsel* OR “cyber-counsel*” OR “online treat*” OR “online therap*” OR “online intervention*” OR “online prevention*” OR “online counsel*” OR “text-messag*” OR textmessag* OR SMS OR texting* OR “short message service*” OR mobile* OR smartphone* OR “cell-phone*” OR cellphone* OR “cellular phone*” OR blended* OR “software app*” OR “handheld device*” OR “hand held device*” OR iPad* OR iPhone* OR email* OR “e-mail*” OR sensor* OR wearable* OR “social media*” OR “social network*” OR “e-counsel*” OR ecounsel* OR palmtop* OR telephone* OR WhatsApp OR Twitter OR Facebook OR Instagram OR forum OR chat* OR “virtual reality*” OR avatar* OR “Conversational agent*” OR “virtual coach” OR “virtual agent*” OR “embodied agent*” OR “relational agent*” OR “interactive agent*” OR “virtual character*” OR “virtual human*” OR “virtual assistant*” OR VR OR “serious game*” OR “serious gaming” OR gamification OR “counseling” OR “counselling”) OR KW(Psychoanalys* OR treatment* OR psychotherap* OR therap* OR “Eye Movement Desensitization Reprocessing” OR EMDR OR intervent* OR imagery OR "cognitive behavio*" OR “cognitive remediation*” OR CBT OR “mindfulness*” OR “self-compassion*” OR “schema focused” OR “trauma focused” OR mantra OR “maudsley model” OR “CBT-E” OR “enhanced cogniti*” OR “Kognitive Verhaltenstherap*” OR KGT OR exposur* OR telehealth OR “tele-health” OR telepsychology OR “tele-psychology” OR telepsychiatry OR “tele-psychiatry” OR “tele-therap*” OR teletherap* OR “tele-medicine” OR telemedicine OR telecare OR “tele-care” OR telecommunicat* OR “tele-communicat*” OR teleconference* OR “tele-conferenc*” OR videoconferenc* OR “video-conferenc*” OR computer* OR electronic* OR digital* OR ehealth OR “e-health” OR “e-treat*” OR “e-therap*” OR mhealth OR “m-health” OR “internet-based*” OR “internet treat*” OR “internet intervention*” OR “internet counsel*” OR “distance counsel*” OR “web-based*” OR cybercounsel* OR “cyber-counsel*” OR “online treat*” OR “online therap*” OR “online intervention*” OR “online prevention*” OR “online counsel*” OR “text-messag*” OR textmessag* OR SMS OR texting* OR “short message service*” OR mobile* OR smartphone* OR “cell-phone*” OR cellphone* OR “cellular phone*” OR blended* OR “software app*” OR “handheld device*” OR “hand held device*” OR iPad* OR iPhone* OR email* OR “e-mail*” OR sensor* OR wearable* OR “social media*” OR “social network*” OR “e-counsel*” OR ecounsel* OR palmtop* OR telephone* OR WhatsApp OR Twitter OR Facebook OR Instagram OR forum OR chat* OR “virtual reality*” OR avatar* OR “Conversational agent*” OR “virtual coach” OR “virtual agent*” OR “embodied agent*” OR “relational agent*” OR “interactive agent*” OR “virtual character*” OR “virtual human*” OR “virtual assistant*” OR VR OR “serious game*” OR “serious gaming” OR gamification OR “counseling” OR “counselling”) | Search modes - Boolean/Phrase | 1,903,869 |
| S2 | DE "Complex Trauma" OR DE "Emotional Trauma" OR DE "Posttraumatic Stress" OR DE "Complex PTSD" OR DE "DESNOS" OR DE "Posttraumatic Stress Disorder" OR DE "Stress and Trauma Related Disorders" OR DE "Psychological Stress" OR TI(“complex trauma*” OR “posttraumatic stress*” OR “post-traumatic stress*” OR “posttraumatic-disorder*” OR “post-traumatic disorder*” OR "post traumatic neuros*" OR "posttraumatic neuros*" OR “traumatic distress*” OR "posttraumatic psychos*" OR "post-traumatic psychos*" OR "posttraumatic syndrom*" OR "post-traumatic syndrom*" OR "trauma-and-stressor-related-disorder*" OR “traumatic stress disorder*” OR ptsd* OR ptss* OR “combat-disorder*” OR “combat-fatigue*” OR “combat-stress*” OR “shell-shock*” OR “combat-neuros*” OR “war-neuros*” OR “battle-fatigue*” OR “psychological stress*” OR “psychological trauma*” OR psychotrauma* OR “psycho-trauma*” OR “trauma-focused*” OR “psychological distress*” OR “emotional trauma*” OR “emotional distress*” OR “emotional damag*” OR “emotional injur*” OR “mental damag*” OR “mental harm*” OR “mental injur*” OR “mental trauma*” OR “sexual trauma*” OR “psychological damag*” OR “psychological harm*” OR “psychological injur*” OR “Posttraumatische Belastungsstörung” OR PTBS) OR AB(“complex trauma*” OR “posttraumatic stress*” OR “post-traumatic stress*” OR “posttraumatic-disorder*” OR “post-traumatic disorder*” OR "post traumatic neuros*" OR "posttraumatic neuros*" OR “traumatic distress*” OR "posttraumatic psychos*" OR "post-traumatic psychos*" OR "posttraumatic syndrom*" OR "post-traumatic syndrom*" OR "trauma-and-stressor-related-disorder*" OR “traumatic stress disorder*” OR ptsd* OR ptss* OR “combat-disorder*” OR “combat-fatigue*” OR “combat-stress*” OR “shell-shock*” OR “combat-neuros*” OR “war-neuros*” OR “battle-fatigue*” OR “psychological stress*” OR “psychological trauma*” OR psychotrauma* OR “psycho-trauma*” OR “trauma-focused*” OR “psychological distress*” OR “emotional trauma*” OR “emotional distress*” OR “emotional damag*” OR “emotional injur*” OR “mental damag*” OR “mental harm*” OR “mental injur*” OR “mental trauma*” OR “sexual trauma*” OR “psychological damag*” OR “psychological harm*” OR “psychological injur*” OR “Posttraumatische Belastungsstörung” OR PTBS) OR KW(“complex trauma*” OR “posttraumatic stress*” OR “post-traumatic stress*” OR “posttraumatic-disorder*” OR “post-traumatic disorder*” OR "post traumatic neuros*" OR "posttraumatic neuros*" OR “traumatic distress*” OR "posttraumatic psychos*" OR "post-traumatic psychos*" OR "posttraumatic syndrom*" OR "post-traumatic syndrom*" OR "trauma-and-stressor-related-disorder*" OR “traumatic stress disorder*” OR ptsd* OR ptss* OR “combat-disorder*” OR “combat-fatigue*” OR “combat-stress*” OR “shell-shock*” OR “combat-neuros*” OR “war-neuros*” OR “battle-fatigue*” OR “psychological stress*” OR “psychological trauma*” OR psychotrauma* OR “psycho-trauma*” OR “trauma-focused*” OR “psychological distress*” OR “emotional trauma*” OR “emotional distress*” OR “emotional damag*” OR “emotional injur*” OR “mental damag*” OR “mental harm*” OR “mental injur*” OR “mental trauma*” OR “sexual trauma*” OR “psychological damag*” OR “psychological harm*” OR “psychological injur*” OR “Posttraumatische Belastungsstörung” OR PTBS) | Search modes - Boolean/Phrase | 115,165 |
| S1 | DE "Underweight" OR DE "Anorexia Nervosa" OR TI(“anorexi*” OR “anorectic” OR “underweight*”) OR AB(“anorexi*” OR “anorectic” OR “underweight*”) OR KW(“anorexi*” OR “anorectic” OR “underweight*”) | Search modes - Boolean/Phrase | 22,218 |

Key: DE= Descriptors,TI= title, AB= abstract and KW= Searches for keywords in the uncontrolled content description of the document

**Web of Science**

| Nr. | Query | Results |
| --- | --- | --- |
| #4 | #1 AND #2 AND #3 | 457 |
| #3 | TS=(Psychoanalys* OR treatment* OR psychotherap* OR therap* OR “Eye Movement Desensitization Reprocessing” OR EMDR OR intervent* OR imagery OR "cognitive behavio*" OR “cognitive remediation*” OR CBT OR “mindfulness*” OR “self-compassion*” OR “schema focused” OR “trauma focused” OR mantra OR “maudsley model” OR “CBT-E” OR “enhanced cogniti*” OR “Kognitive Verhaltenstherap*” OR KGT OR exposur* OR telehealth OR “tele-health” OR telepsychology OR “tele-psychology” OR telepsychiatry OR “tele-psychiatry” OR “tele-therap*” OR teletherap* OR “tele-medicine” OR telemedicine OR telecare OR “tele-care” OR telecommunicat* OR “tele-communicat*” OR teleconference* OR “tele-conferenc*” OR videoconferenc* OR “video-conferenc*” OR computer* OR electronic* OR digital* OR ehealth OR “e-health” OR “e-treat*” OR “e-therap*” OR mhealth OR “m-health” OR “internet-based*” OR “internet treat*” OR “internet intervention*” OR “internet counsel*” OR “distance counsel*” OR “web-based*” OR cybercounsel* OR “cyber-counsel*” OR “online treat*” OR “online therap*” OR “online intervention*” OR “online prevention*” OR “online counsel*” OR “text-messag*” OR textmessag* OR SMS OR texting* OR “short message service*” OR mobile* OR smartphone* OR “cell-phone*” OR cellphone* OR “cellular phone*” OR blended* OR “software app*” OR “handheld device*” OR “hand held device*” OR iPad* OR iPhone* OR email* OR “e-mail*” OR sensor* OR wearable* OR “social media*” OR “social network*” OR “e-counsel*” OR ecounsel* OR palmtop* OR telephone* OR WhatsApp OR Twitter OR Facebook OR Instagram OR forum OR chat* OR “virtual reality*” OR avatar* OR “Conversational agent*” OR “virtual coach” OR “virtual agent*” OR “embodied agent*” OR “relational agent*” OR “interactive agent*” OR “virtual character*” OR “virtual human*” OR “virtual assistant*” OR VR OR “serious game*” OR “serious gaming” OR gamification OR “counseling” OR “counselling”) | 14,165,738 |
| #2 | TS=(“complex trauma*” OR “posttraumatic stress*” OR “post-traumatic stress*” OR “posttraumatic-disorder*” OR “post-traumatic disorder*” OR "post traumatic neuros*" OR "posttraumatic neuros*" OR “traumatic distress*” OR "posttraumatic psychos*" OR "post-traumatic psychos*" OR "posttraumatic syndrom*" OR "post-traumatic syndrom*" OR "trauma-and-stressor-related-disorder*" OR “traumatic stress disorder*” OR ptsd* OR ptss* OR “combat-disorder*” OR “combat-fatigue*” OR “combat-stress*” OR “shell-shock*” OR “combat-neuros*” OR “war-neuros*” OR “battle-fatigue*” OR “psychological stress*” OR “psychological trauma*” OR psychotrauma* OR “psycho-trauma*” OR “trauma-focused*” OR “psychological distress*” OR “emotional trauma*” OR “emotional distress*” OR “emotional damag*” OR “emotional injur*” OR “mental damag*” OR “mental harm*” OR “mental injur*” OR “mental trauma*” OR “sexual trauma*” OR “psychological damag*” OR “psychological harm*” OR “psychological injur*” OR “Posttraumatische Belastungsstörung” OR PTBS) | 159,288 |
| #1 | TS=(“anorexi*” OR “anorectic” OR “underweight*”) | 65,370 |

Key: TS = topic, which includes title, abstract, author keywords and Web of Science Keywords Plus

**Scopus**

**Onderkant formulier**

| History Count | Search Terms | Results |
| --- | --- | --- |
| 4 | #1 AND #2 AND #3 | 988 |
| 3 | TITLE-ABS-KEY ( psychoanalys*  OR  treatment*  OR  psychotherap*  OR  therap*  OR  "Eye Movement Desensitization Reprocessing"  OR  emdr  OR  intervent*  OR  imagery  OR  "cognitive behavio*"  OR  "cognitive remediation*"  OR  cbt  OR  "mindfulness*"  OR  "self-compassion*"  OR  "schema focused"  OR  "trauma focused"  OR  mantra  OR  "maudsley model"  OR  "CBT-E"  OR  "enhanced cogniti*"  OR  "Kognitive Verhaltenstherap*"  OR  kgt  OR  exposur*  OR  telehealth  OR  "tele-health"  OR  telepsychology  OR  "tele-psychology"  OR  telepsychiatry  OR  "tele-psychiatry"  OR  "tele-therap*"  OR  teletherap*  OR  "tele-medicine"  OR  telemedicine  OR  telecare  OR  "tele-care"  OR  telecommunicat*  OR  "tele-communicat*"  OR  teleconference*  OR  "tele-conferenc*"  OR  videoconferenc*  OR  "video-conferenc*"  OR  computer*  OR  electronic*  OR  digital*  OR  ehealth  OR  "e-health"  OR  "e-treat*"  OR  "e-therap*"  OR  mhealth  OR  "m-health"  OR  "internet-based*"  OR  "internet treat*"  OR  "internet intervention*"  OR  "internet counsel*"  OR  "distance counsel*"  OR  "web-based*"  OR  cybercounsel*  OR  "cyber-counsel*"  OR  "online treat*"  OR  "online therap*"  OR  "online intervention*"  OR  "online prevention*"  OR  "online counsel*"  OR  "text-messag*"  OR  textmessag*  OR  sms  OR  texting*  OR  "short message service*"  OR  mobile*  OR  smartphone*  OR  "cell-phone*"  OR  cellphone*  OR  "cellular phone*"  OR  blended*  OR  "software app*"  OR  "handheld device*"  OR  "hand held device*"  OR  ipad*  OR  iphone*  OR  email*  OR  "e-mail*"  OR  sensor*  OR  wearable*  OR  "social media*"  OR  "social network*"  OR  "e-counsel*"  OR  ecounsel*  OR  palmtop*  OR  telephone*  OR  whatsapp  OR  twitter  OR  facebook  OR  instagram  OR  forum  OR  chat*  OR  "virtual reality*"  OR  avatar*  OR  "Conversational agent*"  OR  "virtual coach"  OR  "virtual agent*"  OR  "embodied agent*"  OR  "relational agent*"  OR  "interactive agent*"  OR  "virtual character*"  OR  "virtual human*"  OR  "virtual assistant*"  OR  vr  OR  "serious game*"  OR  "serious gaming"  OR  gamification  OR  "counseling"  OR  "counselling" ) | 25,928,777 |
| 2 | TITLE-ABS-KEY ( "complex trauma*"  OR  "posttraumatic stress*"  OR  "post-traumatic stress*"  OR  "posttraumatic-disorder*"  OR  "post-traumatic disorder*"  OR  "post traumatic neuros*"  OR  "posttraumatic neuros*"  OR  "traumatic distress*"  OR  "posttraumatic psychos*"  OR  "post-traumatic psychos*"  OR  "posttraumatic syndrom*"  OR  "post-traumatic syndrom*"  OR  "trauma-and-stressor-related-disorder*"  OR  "traumatic stress disorder*"  OR  ptsd*  OR  ptss*  OR  "combat-disorder*"  OR  "combat-fatigue*"  OR  "combat-stress*"  OR  "shell-shock*"  OR  "combat-neuros*"  OR  "war-neuros*"  OR  "battle-fatigue*"  OR  "psychological stress*"  OR  "psychological trauma*"  OR  psychotrauma*  OR  "psycho-trauma*"  OR  "trauma-focused*"  OR  "psychological distress*"  OR  "emotional trauma*"  OR  "emotional distress*"  OR  "emotional damag*"  OR  "emotional injur*"  OR  "mental damag*"  OR  "mental harm*"  OR  "mental injur*"  OR  "mental trauma*"  OR  "sexual trauma*"  OR  "psychological damag*"  OR  "psychological harm*"  OR  "psychological injur*"  OR  "Posttraumatische Belastungsstörung"  OR  ptbs ) | 167,693 |
| 1 | TITLE-ABS-KEY ( "anorexi*"  OR  "anorectic"  OR  "underweight*" ) | 134,162 |

Key: ABS, TITLE, AUTHKEY searches in abstract, title and author supplied keywords

**Cochrane CENTRAL**

| ID | Search | Hits |
| --- | --- | --- |
| #1 | (anorexi* OR anorectic OR underweight*):ti,ab,kw | 7709 |
| #2 | (“complex trauma” “complex traumas” OR “posttraumatic stress” OR “post traumatic stress” OR “posttraumatic disorder” OR “posttraumatic disorders” OR “post traumatic disorder” OR “post traumatic disorders” OR "post traumatic neurose" OR “post traumatic neuroses” OR "posttraumatic neurose" OR “posttraumatic neuroses” OR “traumatic distress” OR "posttraumatic psychose" OR “posttraumatic psychoses” OR "post traumatic psychose" OR “post traumatic psychoses” OR "posttraumatic syndrome" OR “posttraumatic syndrome” OR "post-traumatic syndrome" OR “post-traumatic syndromes” OR "trauma and stressor related disorder" OR “trauma and stressor related disorders” OR “traumatic stress disorder” OR “traumatic stress disorders” OR ptsd* OR ptss* OR “combat disorder” OR “combat disorders” OR “combat fatigue” OR “combat stress” OR “shell shock” OR “combat neurose” OR “combat neuroses” OR “war neurose” OR “war neuroses” OR “battle fatigue” OR “psychological stress” OR “psychological trauma” OR “psychological traumas” OR psychotrauma* OR “psycho-trauma” OR “trauma-focused” OR “psychological distress” OR “emotional trauma” OR “emotional traumas” OR “emotional distress” OR “emotional damage” OR “emotional injury” OR “mental damage” OR “mental harm” OR “mental injury” OR “mental trauma” OR “mental traumas” OR “sexual trauma” OR “psychological damage” OR “psychological harm” OR “psychological injury” OR “Posttraumatische Belastungsstörung” OR PTBS):ti,ab,kw | 14911 |
| #3 | (Psychoanalys* OR treatment* OR psychotherap* OR therap* OR “Eye Movement Desensitization Reprocessing” OR EMDR OR intervent* OR imagery OR "cognitive behaviour" OR “cognitive behavior” OR “cognitive behavioural” OR “cognitive behavioral” OR “cognitive remediation” OR CBT OR mindfulness OR “self compassion” OR “schema focused” OR “trauma focused” OR mantra OR “maudsley model” OR “CBT E” OR “enhanced cognitive” OR “Kognitive Verhaltenstherapie” OR KGT OR exposur* OR telehealth OR “tele health” OR telepsychology OR “tele psychology” OR telepsychiatry OR “tele psychiatry” OR “tele therapy” OR “tele therapies” OR teletherap* OR “tele medicine” OR telemedicine OR telecare OR “tele care” OR telecommunicat* OR “tele communication” OR teleconference* OR “tele conference” OR “tele conferencing” OR videoconferenc* OR “video conference” OR “video conferencing” OR computer* OR electronic* OR digital* OR ehealth OR “e health” OR “e treatment” OR “e therapy” OR “e therapies” OR mhealth OR “m health” OR “internet based” OR “internet treatment” OR “internet intervention” OR “internet counseling” OR “distance counseling” OR “web based” OR cybercounsel* OR “cyber counseling” OR “online treatment” OR “online therapy” OR “online intervention” OR “online prevention” OR “online counseling” OR “text message” OR “text messages” OR textmessag* OR SMS OR texting* OR “short message service” OR “short message services” OR mobile* OR smartphone* OR “cell phone” OR “cell phones” OR cellphone* OR “cellular phone” OR “cellular phones” OR blended* OR “software app” OR “software apps” OR “handheld device” OR “handheld devices” OR “hand held device” OR “hand held devices” OR iPad* OR iPhone* OR email* OR “e mail” OR “e mails” OR sensor* OR wearable* OR “social media” OR “social network” OR “social networks” OR “e counseling” OR ecounsel* OR palmtop* OR telephone* OR WhatsApp OR Twitter OR Facebook OR Instagram OR forum OR chat* OR “virtual reality” OR avatar* OR “Conversational agent” OR “conversational agents” OR “virtual coach” OR “virtual agent” OR “virtual agents” OR “embodied agent” OR “embodied agents” OR “relational agent” OR “relational agents” OR “interactive agent” OR “interactive agents” OR “virtual character” OR “virtual characters” OR “virtual human” OR “virtual humans” OR “virtual assistant” OR “virtual assistants” OR VR OR “serious game” OR “serious games” OR “serious gaming” OR gamification OR counseling OR counselling):ti,ab,kw | 1502457 |
| #4 | #1 AND #2 AND #3 | 57 |
| #5 | #4 AND Trials | 55 |

Key: ti,ab,kw searches in title, abstract and author supplied keywords
